# Arrhythmic Mitral Valve Prolapse: Introducing an Era of Multimodality Imaging-Based Diagnosis and Risk Stratification

**DOI:** 10.3390/diagnostics11030467

**Published:** 2021-03-08

**Authors:** Deni Kukavica, Marco Guglielmo, Andrea Baggiano, Giuseppe Muscogiuri, Laura Fusini, Manuela Muratori, Gloria Tamborini, Valentina Mantegazza, Alessandro Trancuccio, Carlo Arnò, Andrea Mazzanti, Mauro Pepi, Silvia Giuliana Priori, Gianluca Pontone

**Affiliations:** 1 Molecular Cardiology, Istituti Clinici Scientifici Maugeri IRCCS, 27100 Pavia, Italy; deni.kukavica01@universitadipavia.it (D.K.); alessandro.trancuccio01@universitadipavia.it (A.T.); carlo.arno01@universitadipavia.it (C.A.); andrea.mazzanti@icsmaugeri.it (A.M.); silvia.priori@icsmaugeri.it (S.G.P.); 2 Department of Molecular Medicine, University of Pavia, 27100 Pavia, Italy; 3 Department of Cardiovascular Imaging, Centro Cardiologico Monzino IRCCS, 20138 Milan, Italy; Marco.Guglielmo@cardiologicomonzino.it (M.G.); Andrea.Baggiano@cardiologicomonzino.it (A.B.); Giuseppe.Muscogiuri@cardiologicomonzino.it (G.M.); Laura.Fusini@cardiologicomonzino.it (L.F.); Manuela.Muratori@cardiologicomonzino.it (M.M.); Gloria.Tamborini@cardiologicomonzino.it (G.T.); Valentina.Mantegazza@cardiologicomonzino.it (V.M.); Mauro.Pepi@cardiologicomonzino.it (M.P.); 4 Molecular Cardiology, Fundación Centro Nacional de Investigaciones Cardiovasculare, 28029 Madrid, Spain

**Keywords:** mitral valve prolapse, sudden cardiac death, arrhythmia, risk factors, multimodality imaging, echocardiography, cardiovascular magnetic resonance

## Abstract

Mitral valve prolapse is a common cardiac condition, with an estimated prevalence between 1% and 3%. Most patients have a benign course, but ever since its initial description mitral valve prolapse has been associated to sudden cardiac death. Although the causal relationship between mitral valve prolapse and sudden cardiac death has never been clearly demonstrated, different factors have been implicated in arrhythmogenesis in patients with mitral valve prolapse. In this work, we offer a comprehensive overview of the etiology and the genetic background, epidemiology, pathophysiology, and we focus on the state-of-the-art imaging-based diagnosis of mitral valve prolapse. Going beyond the classical, well-described clinical factors, such as young age, female gender and auscultatory findings, we investigate multimodality imaging features, such as alterations of anatomy and function of the mitral valve and its leaflets, the structural and contractile anomalies of the myocardium, all of which have been associated to sudden cardiac death.

## 1. Introduction

### 1.1. Mitral Valve Anatomy

**Figure 1:**
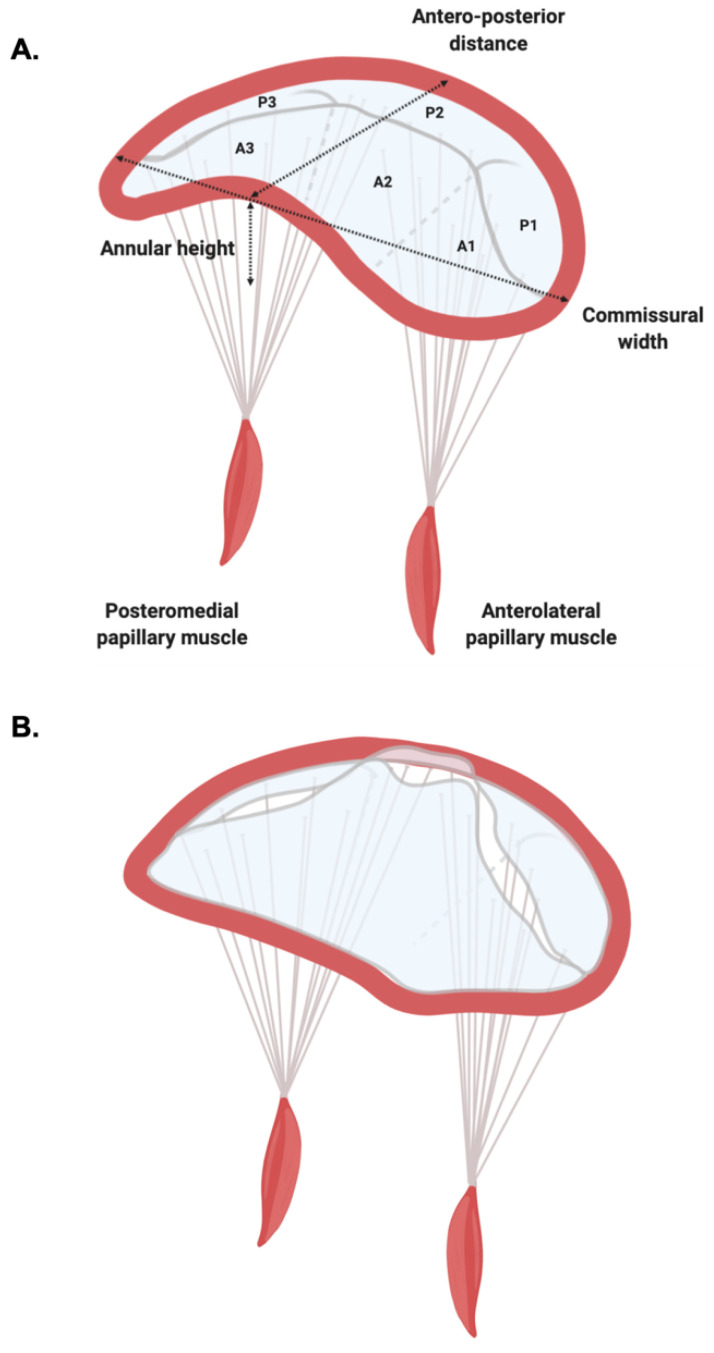
Mitral Valve Anatomy. (**A**) Normal valvular anatomy, with the saddle-shaped annulus and two leaflets, divided according to the Carpentier classification. (**B**) Mitral valve prolapse is characterized by superior displacement of one or both leaflets into the left atrium. Other than the prolapse, the mitral valve undergoes profound changes in systole with the flattening of the annulus and the increase of posterior circumference.

Mitral valve (MV) is a complex three-dimensional (3D) structure composed of valvular (i.e., annulus, commissures and leaflets) and sub-valvular (i.e., papillary muscles and chordae tendineae) components (Figure 1).

The pivotal study by Levine and colleagues demonstrated that the mitral annulus (MA) is a saddle-shaped structure with its most apical points being located medially and laterally, while its most basal points are located anteriorly and posteriorly [1] (Figure 1). Later studies demonstrated that the shape and dimensions of MA vary dynamically with the cardiac cycle [2]: In diastole, it is more circular in shape, while in systole it bends becoming more saddle-shaped [3]. The saddle-shape is highly conserved across species [4] and is thought to confer a mechanical advantage to the leaflets by reducing peak leaflet stress [5].

The motion of the MA is passive and is functionally related to the contraction-relaxation cycle of adjacent atrial and ventricular musculature, as well as the motion of the aortic root [6]. During systole, the MA moves apically, with the anterior portion moving posteriorly and the posterior portion moving anteriorly [6].

The leaflets of the MV differ: The mural (posterior) leaflet is narrower and presents indentations forming three segments; while the aortic (anterior) leaflet is broader, semi-circular, and presents two distinct zones (a rough and a clear one) [7]. The broadly used Carpentier classification refers to the three segments of posterior leaflet as P1, P2 and P3 (laterally to medially), and the corresponding, non-anatomical segments of the anterior leaflet as A1, A2 and A3 [8] (Figure 1).

The classical work of Lam and colleagues defined the anatomy of the sub-valvular apparatus. They found that chordae tendineae arise from the tips of the papillary muscles (anterolateral and posteromedial) and depending on the site of insertion can be classified as primary (those that attach to the distal, free edge of the rough zone), secondary (those that ventricular surface of the body of the leaflet) and tertiary (those exclusive to the posterior leaflet, attaching to the ventricular wall) [9]. The posteromedial papillary muscle gives rise to chords attaching to the medial half of both leaflets (i.e., posteromedial commissure, P3, A3, and medial portions of P2 and A2), while the anterolateral papillary muscle gives rise to chords inserting into the lateral portion of the leaflets (i.e., anterolateral commissure, A1, P1, and lateral segments of P2 and A2) [7] (Figure 1).

### 1.2. Mitral Valve Prolapse

Mitral valve prolapse (MVP) is a spectrum of clinical entities characterized by superior displacement of one or both leaflets into the left atrium (LA) [10] (Figure 1).

At the present time, MVP is commonly classified as primary (i.e., non-syndromic) or secondary (i.e., syndromic). Familial studies have suggested that heritability is common [11], and thus, primary MVP can be further subdivided into sporadic and familial forms.

The initial hypothesis of X-linked inheritance had been put forth in the late 1960s [12], but almost forty years passed before the identification of a missense filamin C (*FLNC*) mutation (p. Pro637Gln) as the first MVP gene in a large French Caucasian family [13]. These initial observations were further supported by the identification of three *FLNC* mutations (two missense and one large deletion) in three unrelated families (British Caucasian, Black African and Hong Kong Chinese) [13].

Additionally, linkage studies identified three loci with an autosomal dominant inheritance: *MMVP1* [14], *MMVP2* [15] and *MMVP3* [16] mapping to chromosomes 16p12.1-p11.2, 11p15.4 and 13q31.3-q32.1, respectively. Despite solid evidence backing the heritability, the genes responsible remained largely elusive. Hitherto, only missense variants on *DCHS1* gene [17] coding for dachsous homolog 1 and mapping to the *MMVP2* locus, have been described in two non-related families with a strong family history of MVP.

**Figure 2:**
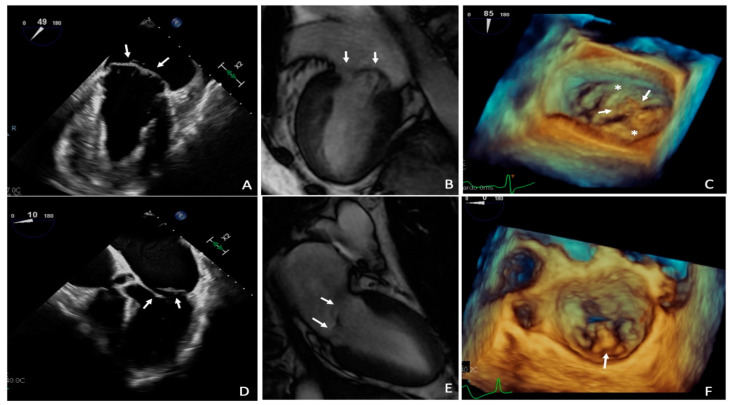
Multimodality assessment of MVP due to Barlow’s disease and due to fibroelastic deficiency; 2D TEE (**A**) and CMR (**B**) images show thickening and prolapse of both mitral valve leaflets in Barlow’s disease; 3D TEE (**C**) confirms mitral prolapse involving multiple segments of both leaflets (asterisks). Two ruptured chordae (arrows) are also visible; 2D TEE (**D**) and CMR (**E**) show thin mitral valve leaflets (arrows) with posterior leaflet flail confirmed by 3DTEE ((**F**), arrow) in fibroelastic deficiency. CMR: Cardiac Magnetic Resonance; FED: Fibroelastic deficiency; TEE: Transeoesophageal echocardiography.

Secondary MVP is typically associated to a range of connective tissue disorders such as Marfan syndrome [18], Loeys-Dietz syndrome [19], Ehler-Danlos syndrome [20], and osteogenesis imperfecta [21]. Pathologically, MVP can be divided into Barlow’s disease and fibroelastic deficiency (Figure 2). Barlow’s disease is a characterized by myxoid infiltration causing excessive, thickened and distended leaflet tissue resulting frequently in multi-segmental prolapse [22]. Fibroelastic deficiency, as the name suggests, results from the impaired production of connective tissue, with deficiency of collagen, elastins, and proteoglycans, causing leaflet and chordae thinning, which may rupture [22].

## 2. Mitral Valve Prolapse: A Historical Perspective

Initial description of MVP had been provided by Barlow and Bosman in 1966 as an “auscultatory-electrocardiographic syndrome” [23]: Apical late systolic murmur coupled with a systolic click; inverted T wave in infero-lateral leads and mild mitral regurgitation (MR). The same year, Criley and colleagues confirmed the association of late systolic murmur and systolic prolapse of the posterior leaflet of the MV with MR, coining the term MVP and suggesting that it might be associated with a pathology of the chordae tendineae [24]. The seminal work of Barlow and colleagues provided morphological evidence for this hypothesis and established MVP as a specific syndrome [25].

In the early days of echocardiography, extremely high prevalence rates have been identified, with up to a third of healthy children being affected [26]. One of the principal factors driving such high diagnostic rates had been the erroneous assumption that the MV is a Euclidian plane during the entire cardiac cycle [27], which led to considering any leaflet displacement above the presumably planar annular plane as sufficient evidence for diagnosis. Levine and colleagues redefined the understanding of the MV anatomy, establishing that it is saddle-shaped [1], with major implications for both the epidemiology and diagnosis of MVP.

MVP is a considered a common condition, with an estimated community-based prevalence between 1% [11] and 2.4% [28] in Caucasian population, 0.5% in Black population [29], 1.7% in American Indian population [30], 2.2% in Chinese population [31], 2.7% in South Asian population [31] using the standardized echocardiographic diagnostic criteria. Moreover, MVP represents the most common cause of primary MR in the Western world [32].

Since the first description, MVP had been associated to ventricular arrhythmias (VA) [23] and sudden cardiac death (SCD) [25], but has been generally considered a benign entity. The work of Nishimura and associates identified a subset of patients who were at much higher risk for SCD [33], later termed “malignant MVP” or “arrhythmic MVP”, thus paving the way for the risk stratification for SCD in patients with MVP.

## 3. Diagnosis of MVP

Cardiac imaging represents the gold standard for the diagnosis of MVP, and has largely supplanted the auscultatory signs, with most diagnoses being made using echocardiography.

### 3.1. Echocardiography

**Figure 3:**
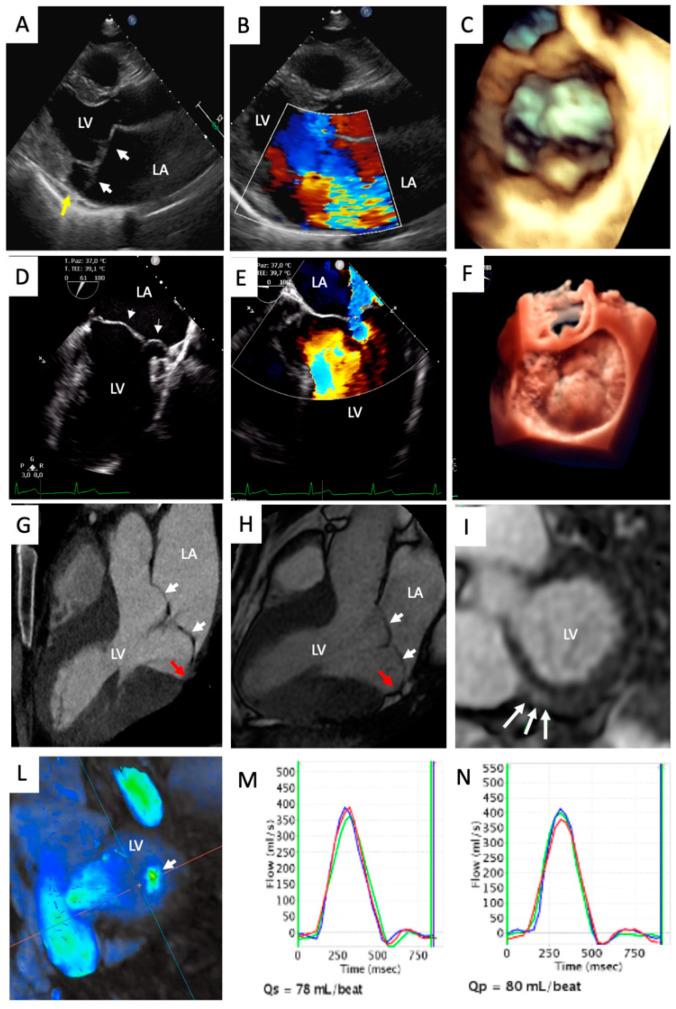
Multimodality MVP assessment in a 37-year-old male symptomatic for exercise dyspnea. (**A**) TTE PLAX view showing a myxomatous MV with bileaflet MVP (white arrows) with associated MAD (yellow arrow); (**B**) ColorDoppler TTE PLAX view showing centrovalvular mitral regurgitation jet; (**C**) 3D-TTE demonstrating bileaflet MVP; (**D**) bicommisural TEE images confirming bileaflet MVP (white arrows); (**E**) TEE 4-Ch view, assessment of mitral regurgitation severity measuring the PISA radius; (**F**) 3D-TEE with photo-realistic 3D rendering confirming bileaflet MVP; (**G**,**H**) CCT (**G**) and CMR (**H**) confirming the presence of bileaflet MVP (white arrows) with associated MAD (red arrow); (**I**) CMR late gadolinium enhancement images showing focal fibrosis of the LV inferobasal wall; (**L**) four-dimensional (4D)-flow CMR basal short axis view showing centrovalvular mitral regurgitant jet; (**M**,**N**) phase contrast measurements of aortic (**M**) and pulmonary (**N**) flow. CCT: Cardiac CT; CMR: Cardiac Magnetic Resonance; MV: Mitral Valve; MAD: Mitral Annular Disjunction; MVP: Mitral Valve Prolapse; PISA: Proximal Isovelocity Surface Area; PLAX: Parasternal Long Axis; TEE: Transesophageal echocardiography; TTE: Transthoracic Echocardiography.

The 2013 European Society of Cardiology recommendations define the MVP as an abnormal systolic displacement of one or both mitral leaflets more than 2 mm below the annular plane, superiorly into the left atrium, as seen in the parasternal or eventually the apical long-axis view [34] on two-dimensional trans-thoracic echocardiography (2D TTE) (Figure 3). Importantly, apical four-chamber view should not be used for diagnosis. As mentioned in the introduction, MV is a saddle-shaped structure with its most apical points being located medially and laterally, while its most basal points are located anteriorly and posteriorly [1]. Therefore, leaflets may appear to ascend above the MA in the apical four-chamber view without leaflet distortion or actual displacement above the entire MV [1], leading to misdiagnosis.

In 2006, the data from this laboratory, in a cohort of 112 patients with an established diagnosis of severe MR due to degenerative MVP, demonstrated that trans-esophageal echocardiography (TEE) provides the most accurate localization of pathology, assuming surgical findings as reference (87% and 95.6% of cases, using 2D and 3D TEE), and should be considered the gold standard for surgical planning [35] (Figure 3). Independent studies confirmed the finding that 3D TEE was more accurate (92–100%) than 2D TEE (80–96%) in identification of prolapsed segments and was comparable with direct surgical measures [36]. Interestingly, a recent study from our group suggests that 3D methods are superior to 2D methods: 3D TTE was superior to 2D TEE in the recognition and localization of MVP (93% vs. 91%) [37].

The diagnosis of MVP must always be accompanied with an extensive research for the potential presence of MR, and ultimately, a robust quantification of its severity (Figure 3). Echocardiography represents the most common technique used for the quantification of the severity of MR [38] and relies on the integration of qualitative, semi-qualitative and quantitative parameters. Careful assessment of the valvular morphology together with the qualitative assessment of the color Doppler (jet size, morphology and extent) and continuous Doppler (density and shape) provide initial clues about the severity of the MR [38]. Semi-quantitative features such as vena contracta (>7 mm), systolic pulmonary vein flow reversal, E wave dominance at mitral inflow pulsed Doppler (E > 1.2 m/s) are all consistent with severe MR [38]. Both the 2017 ESC Guidelines [38] and the 2020 ACC/AHA Guidelines [39] agree that an effective regurgitant orifice area (EROA) greater or equal to 40 mm^2^ and regurgitant volume greater or equal to 60 mL/beat are consistent for severe MR. Lastly, a novel and complementary method for the identification of severe MR that has been suggested recently is the average pixel intensity method, with a cut-off greater than 125 au proposed for the identification of severe MR [40].

Importantly, both regional and global LV systolic function should always be evaluated using echocardiography, according to the 2017 ESC Guidelines [38].

### 3.2. Cardiovascular Magnetic Resonance

Cardiovascular magnetic resonance (CMR) is considered a robust noninvasive technique that can provide comprehensive assessment of the MV [41]. The added value of CMR is represented by accurate estimation of left ventricular (LV) volumes and function, as well as multiparametric tissue characterization and important for risk stratification.

The pioneering work of Han and colleagues established that a 2 mm threshold for leaflet excursion into the LA, identical to echocardiography criteria, in the LV outflow tract long-axis view yielded 100% sensitivity and 100% specificity [42] (Figure 3).

Later on, the work by Delling and colleagues identified anterior leaflet length, posterior leaflet displacement, posterior leaflet thickness, and the presence of flail leaflet as the best CMR valvular determinants of MVP-related MR [43].

The 2020 AHA/ACC Guidelines suggest that the use of CMR is appropriate for the assessment of LV volumes, function and MR severity, especially when TTE does not address these issues satisfactorily [39].

In terms of quantitative assessment, CMR planimetry of the EROA has been shown to be feasible and in good agreement with invasive angiographic grading (*r* = 0.84, *p* < 0.0001) and non-invasive echocardiographic assessment (*r* = 0.81, *p* < 0.0001) [44]. Importantly, using an EROA cut-off of 40 mm^2^, CMR detected MR with sensitivity and specificity of 94% and 94%, respectively [44]. CMR also permits an accurate calculation of regurgitant volume, using different techniques: The most widely used one is the difference between the LV stroke volume calculated using planimetry of cine steady state, free-precession images and the aortic (systolic) forward volume obtained by phase-contrast images [41].

Novel techniques such as 4D-flow CMR offer great promise for an accurate qualitative assessment of MR, especially for eccentric jets [41].

The evidence of CMR capability to detect tissue changes in MVP patients was published in recent studies which demonstrated that only patients with MVP had a significant reduction of papillary muscle signal compared to LV parietal myocardium [45]. Interestingly, these findings were not related to the presence (or absence) of fibrosis and allowed the identification of patients with MVP, not only when compared to healthy controls, but even when compared with patients with other causes of MR and compared with patients with hypertrophic cardiomyopathy [45].

### 3.3. Cardiac Computed Tomography

Last, cardiac computed tomography (cCT) is another feasible, but under-investigated method for the diagnosis of MVP. It boasts excellent specificity (95%), but as compared to echocardiography, cCT has a significantly lower sensitivity (80% versus 87%), which is particularly marked in bileaflet MVP [46] (Figure 3).

## 4. Multimodality Imaging Features Associated to Sudden Cardiac Death

In different series, MVP is found in approximately 2–4% of all causes of SCD in the young, both in the general population and in competitive athletes. Albeit SCD has been associated with MVP since the earliest descriptions of the condition [24,33], the potential underlying mechanisms remain incompletely understood [47]. Given the high prevalence of MVP in the general population, the retrospective nature of most series and the absence of genetic testing in almost all series, up to now the association between MVP and SCD remains controversial and under debate. It is possible that at least a proportion of this association may be attributable to ascertainment bias [48], resulting in an overestimated SCD rates.

Demographic parameters, such as young age and female gender [49], as well as auscultatory findings [50] have been frequently used to identify a subset of patients at the highest risk for SCD [47], but there has been much debate on the use of cardiac imaging in risk stratification.

**Figure 4:**
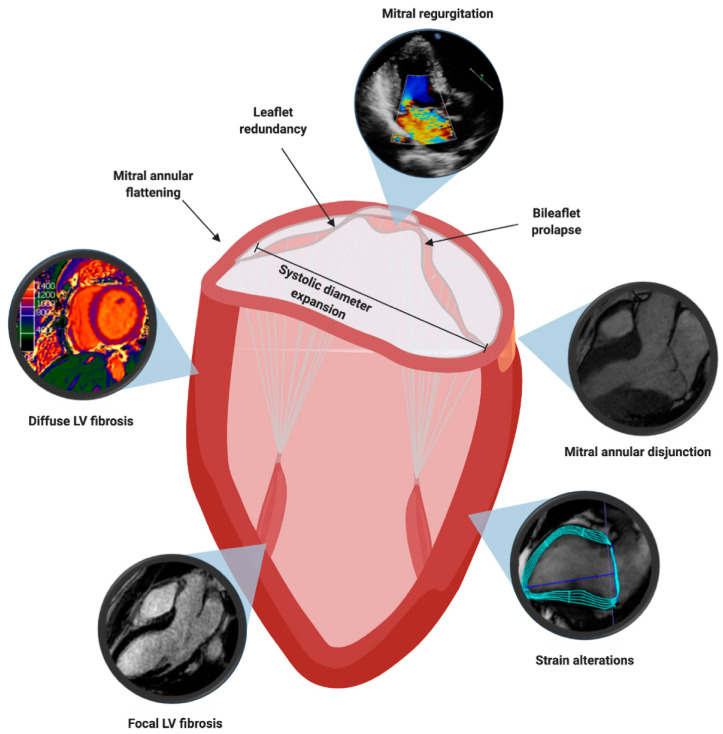
Multimodality Imaging Features Associated to Sudden Cardiac Death in Mitral Valve Prolapse.

In this section, we will offer a deep-dive into four broad, overlapping and non-exclusive groups of imaging-based risk factors: mitral valve leaflet alterations, mitral valve annulus alterations, myocardial structural abnormalities, myocardial contraction alterations; and their interactions with the electrophysiologic risk factors (Figure 4).

### 4.1. Mitral Valve Leaflet Alterations

One of the first series, compiled by Dr. Jeresaty in 1976, described a number of MV characteristics that would later be confirmed as risk factors for SCD in patients with MVP. Namely, in the series of 12 patients (9/12 females (75%)) from a larger cohort of 240 patients who had died suddenly, redundant myxomatous mitral leaflets, mitral regurgitation (86%) and bilateral prolapse (70%) were significantly overrepresented [51].

The first prognostic factor investigated for risk stratification was leaflet redundancy (i.e., thickness of 5 mm or more of one or both mitral leaflets; Figure 4). In the cohort of Nishimura and colleagues, it was an independent risk factor for SCD, with all six patients who died presenting redundant leaflets (6/97 (6.2%) with redundant leaflets vs. 0/140 (0%) without redundant leaflets). Successively, the work of Marks and colleagues defined the patients with MVP and leaflet redundancy as “classic” MVP, while those patients without were termed “non-classic” MVP [52]. Importantly, “classic” MVP has also been demonstrated to be an independent predictor of VA in an Italian cohort [53], a finding later confirmed in larger cohorts [54]; with the incidence of complex VA shown to correlate positively with the degree of MVP [55].

Although most patients with MVP have no MR or mild MR (38% and 46%, respectively) [56], it is estimated that around 7% of patients with MVP [57] have severe MR. Interestingly, the multivariate analysis of the natural history of the disease, conducted in Olmsted County (Minnesota, USA) on a combined endpoint of death and heart failure related to MVP, endocarditis, and mitral surgery, identified severe MR as an independent predictor (Figure 4). Patients with severe MR were found to be at a three-fold increased risk of cardiovascular mortality [56] and although the cardiovascular mortality was partly related to the ventricular dysfunction MR induces, it was mostly independent [56]. This is reinforced by an earlier, independent finding that LV volume overload is associated with a high recurrence rate of VA [58].

Although sporadic reports of bileaflet MVP (Figure 4) associated to SCD continued being reported in the literature for years [59], almost four decades after the first description of the arrhythmic MVP, the data from Ackermann laboratory, who reported an unusually high incidence of bileaflet MVP in a cohort of 24 patients who survived an out-of-hospital cardiac arrest [60], rekindled the interest of the scientific community for the topic.

The work by the Padua group supports the high prevalence of bileaflet MVP in patients with SCD. In their cohort 43/650 (7%) patients died suddenly at an age <40 years of age: In 70% of them, bileaflet involvement was identified [49].

In select cohorts (5 patients), preliminary data seem to hint at the reduction, but not complete abolishment, of the burden of malignant arrhythmias and the rate of appropriate shocks following the surgical correction of bileaflet MVP [61], suggesting that multiple factors might be at play. These findings are supported by the more recent data, suggesting that bileaflet MVP in isolation, despite its association with VA, does not seem to portend a poorer prognosis for SCD at the population level [62].

### 4.2. Mitral Valve Annulus Alterations

Different alterations involving the MA itself have been extensively studied, but have been only indirectly implicated as risk factors for SCD: As precursors [63] for the development of MR and as risk factors for non-sustained VA.

Perhaps the most important aspect of mitral annular dysfunction in MVP is mitral annular disjunction (MAD; Figure 4). MAD is defined as a wide separation between the atrial wall-mitral valve junction and the atrial aspect of the LV free wall [64]. Currently, its diagnosis relies on the separation greater or equal to 5 mm between the mural leaflet insertion into the left atrial wall and the base of the LV free wall, a cut-off deriving from the initial pathological description [64], later verified by TTE studies [65]. CMR is the gold standard for the diagnosis, although TTE has been shown to have good agreement with it [66]. In different works it has been shown to be a common, but not exclusive, feature of patients with MVP, independently from the diagnostic technique used: 2D TTE [65,67], 3D TTE [68], and to correlate to pathological findings [69].

MAD has profound implications on the dynamic behavior of the MV shape and recent works have suggest that it may be both an anatomic feature of the MA itself [70] and an independent syndrome [71]. In patents with MAD and MVP both the annular height and annular height-to-commissural width ratio (Figure 1) decreased, resulting in paradoxical annular flattening during systole [68], while the systolic annular diameter increased [67]. Although annular alterations have not been investigated as a risk factor for SCD, both in vivo [5] and advanced computational [72] evidence demonstrate the significance of the physiological saddle-shaped annulus in maintenance of normal MV leaflet stress, suggesting that MAD might be one of the precursor mechanisms leading to MR in MVP [63] but also for the occurrence of VA.

A correlation between the severity of MAD and the occurrence of VA has been demonstrated: MAD > 8.5 mm was a strong predictor for non-sustained ventricular tachycardia (NSVT) [67]. Considering only the patients with MVP but without MR, MAD remained a predictor of the frequency of premature ventricular complexes (PVCs) and NSVT [50], suggesting that it might be independent from MR. This finding is supported by a recent work by Essayagh and colleagues who found that in a multivariate analysis severe MAD was associated with a sevenfold increased risk of VA [54]. Although associated with MR and NSVTs, MAD has never been tested as an independent risk factor for SCD—large studies using hard-endpoints will give the last word on its relevance for SCD.

### 4.3. Myocardial Structural Abnormalities

Initial observations linking intramyocardial fibrosis to MVP and SCD were made in the histological studies and have been subsequently confirmed by robust CMR studies. Namely, in the work by the Padua group, histological evidence of LV fibrosis had been found in papillary muscles (43/43 SCD victims) and the infero-basal wall (38/43 patients, 88%) [49]. These findings were confirmed in the cohort of living patients of Italian descent, affected by MVP and VA, who had evidence of late gadolinium enhancement (LGE) in 93% (Figure 4).

A mechanicistic insight into the molecular pathophysiology of mechanical stress-induced fibrosis has been offered by Blomme and colleagues in 2019 who showed a transient upregulation of transforming growth factor β2 (TGF-β2) and a longer lasting connective tissue growth factor (CTGF) expression, a known downstream effector of TGF-β [73]. These findings provide a consistent link to the known TGF-β signaling enhancement in a number of connective disorders linked to MVP (i.e., Marfan syndrome [74], Loeyts-Dietz syndrome [75]) and to the previous human expression studies [76].

At odds with these findings, other studies have found much lower rates of LGE, with clear CMR demonstration in only a third of patients [77,78]. Interestingly, one of these studies also reported significantly shorter postcontrast T_1_ times (pT_1_) when compared with controls (334 ± 52 vs. 363 ± 58 ms). Additionally, the same group identified that patients with MVP and VA had longer pT_1_ compared to the patients with MVP and without VA (324 (IQR: 296 to 348 ms) vs. 354 (IQR: 327 to 376 ms)). Our group provided evidence for the prolongation of global native T_1_ times (nT_1_) in patients with MVP compared to healthy controls (1124.9 ± 97.5 ms vs. 1007.4 ± 26.1 ms) [79]. We found that the distribution of nT_1_ prolongation reflected the “typical” sites: nT_1_ times were significantly higher in the basal and mid-LV inferolateral walls [79]. Importantly, our work demonstrated that there was no correlation between MR severity and nT_1_ times, signifying that diffuse fibrosis in patients with MVP (Figure 3 and Figure 4) is likely not a direct consequence of volume overload [79]. The aforementioned radiological findings corroborate the morphological evidence demonstrated in autoptic series, which suggest that SCD can occur even in the absence of macroscopical fibrosis [80], which is detectable by LGE.

These observations suggest that even though macroscopic, focal fibrosis clearly serves as electrophysiological substrate for malignant VA, as evidenced with the highest arrhythmic burden in patients with LGE [77], it is possible that it represents a later stage of the disease process [81]. At the same time, it is likely that diffuse myocardial fibrosis, which is independent from the MR, may contribute to arrhythmia onset even before LGE could be detected.

### 4.4. Myocardial Contraction Abnormalities

In addition to the aforementioned morphological myocardial anomalies, a range of myocardial contraction abnormalities have also been implicated as potential risk factors for SCD in patients with MVP, but no data corroborating these hypotheses exist at the present time.

In an echocardiography study, Muthukumar and colleagues described a high-velocity systolic signal using tissue Doppler imaging (TDI) and coined the term “Pickelhaube sign” [82], for the reminiscence between the pattern and a 19th century Prussian spiked helmet. Although never demonstrated to be a risk factor for SCD, this TDI signal has been found to be overrepresented in a group of patients with arrhythmic MVP [82], and it has been proposed that the sharp tugging of the papillary muscle and the LV wall, which move apically, may be arrhythmogenic, also in the absence of LGE at CMR [83].

Recently published data from this laboratory demonstrated strain alterations using CMR feature tracking (CMR-FT). Patients with MVP showed reduced radial and circumferential strain values in basal and mid-ventricular inferolateral segments, as compared to other segments [79] (Figure 4). Additionally, these sites were the very same sites in which we identified the increase in nT_1_ times, suggesting a nexus between the two [79].

Additional strain-derived indices have been assessed as risk factors, such as mechanical dispersion. Interestingly, mechanical dispersion, was not merely significantly higher in patients with arrhythmic MVP, but also an independent predictor of arrhythmic risk [84].

### 4.5. Electrophysiologic Risk Factors

The baseline electrocardiogram is often characterized by the presence of repolarization abnormalities in the infero-lateral leads (T wave inversion) [23,85] and mild QT interval prolongation [84]. The QT interval prolongation is especially interesting as it might act as a facilitator for one of the proposed electrophysiological mechanisms of arrhythmia origin in patients with MVP: Stretch-induced afterdepolarizations [85,86].

The stretch-induced afterdepolarizations may occur due to the contact of the prolapsing leaflets with the ventricular myocardium during diastole, due to the stretch of the leaflet itself or at the site of the insertion of papillary muscle [87]. Regardless of the site of the origin, stretch-induced afterdepolarization may act as a trigger for VA, especially in a tissue with a pro-arrhythmogenic functional (QTc interval prolongation) and structural substrate (fibrosis) [88].

In fact, a recent meta-analysis demonstrated that PVC-triggered ventricular fibrillation (VF) [89] seems to be the primary arrhythmia in patients with MVP who experienced a cardiac arrest [47]. Although the presence of PVCs per se has never been demonstrated as a risk factor for SCD, the origin of the VA correlates well with the peculiar, reproducible pattern of fibrosis [90] and the electrocardiograms of patients with MVP are compatible with such a distribution. Even though it has been speculated since the 1980s that the arrhythmias due to MVP originate from the infero-basal region of the LV [91], only recently convincing evidence has been put forward to confirm this hypothesis. Two different groups provided a robust characterization of the electrophysiological substrate of arrhythmias in MVP, identifying the papillary muscles as the site of PVC origin in patients with PVC-triggered VF and MVP [83,89].

## 5. Recommendations

The European Society of Cardiology guidelines for the management of ventricular arrhythmias and the prevention of sudden cardiac death do not pose specific indications for the risk stratification in patients with MVP. In fact, a granular risk stratification is still not possible, in the absence of large prospective studies on well-characterized cohorts of patients in primary prevention of sudden cardiac death.

Some experts have suggested that patients with MVP should undergo a focused work-up with 12-lead ECG, exercise stress test, 24-hour ambulatory ECG Holter and an echocardiogram [85]. In the group of patients with risk factors, periodic cardiac magnetic resonance and prolonged electrocardiographic monitoring using implantable loop recorder should be considered [85].

## 6. Conclusions

MVP represents a common cardiac condition, with an estimated prevalence between 1% to 3%. Most patients with MVP have a benign course, but MVP has often been associated to SCD. Notwithstanding the fact that the causal relationship between MVP and SCD has never been clearly documented, the data we have presented suggest that there are different factors which may contribute to arrhythmogenesis in patients with MVP.

Current literature suggests that the alterations of the MV leaflets (leaflet redundancy and bileaflet prolapse) and the MA (mitral annular flattening and MAD), may be interdependent and might represent the real *primum movens* for the occurrence of arrhythmias in patients with MVP. The results of the mechanical dysfunction are multifaceted and are likely to represent a pathophysiological spectrum. Mechanical dysfunction, resulting in sharp tugging of the papillary muscle and the LV wall, can represent an arrhythmogenic trigger, with the electrical mechanism implicated seems to be mediated by stretch-induced afterdepolarizations. The resultant triggered activity may induce PVCs, typically located in the proximity of the papillary muscles and can be the trigger for VF. On the other hand, the aforementioned mechanical dysfunction may arise in mechanical stress-induced fibrosis via a TGF-β-mediated pathway in the long run, which can manifest as either focal or diffuse fibrosis, which in turn act as a pathological substrate. Lastly, an important consequence of MVP is the development of MR, a strong risk factor for SCD.

It is clear how accurate multimodality imaging plays a central role in MVP. Using the variety of techniques at our disposal, we can study the complex valvular anatomy, detecting and quantifying the degree of MVP; assess the presence and severity of MR; identify the myocardial contraction abnormalities; and characterize the myocardial structural abnormalities, such as focal and/or diffuse fibrosis. Given the link between mechanical dysfunction and electrophysiology, imaging represents the most powerful tool at our disposal for the identification of risk factors that have been suggested to predispose patients with MVP to SCD.

## Data Availability

No new data were created or analyzed in this study. Data sharing is not applicable to this article.
